# Enhancer selectivity across cell types delineates three functionally distinct enhancer-promoter regulation patterns

**DOI:** 10.1186/s12864-024-10408-w

**Published:** 2024-05-16

**Authors:** Chengyi Wu, Jialiang Huang

**Affiliations:** 1 State Key Laboratory of Cellular Stress Biology, School of Life Sciences, Faculty of Medicine and Life Sciences, Xiamen University, Xiamen, 361102 Fujian China; 2 National Institute for Data Science in Health and Medicine, Xiamen University, Xiamen, 361102 Fujian China

**Keywords:** Enhancer-promoter interactions, Enhancer selectivity, Gene regulation patterns across cell types

## Abstract

**Background:**

Multiple enhancers co-regulating the same gene is prevalent and plays a crucial role during development and disease. However, how multiple enhancers coordinate the same gene expression across various cell types remains largely unexplored at genome scale.

**Results:**

We develop a computational approach that enables the quantitative assessment of enhancer specificity and selectivity across diverse cell types, leveraging enhancer-promoter (E-P) interactions data. We observe two well-known gene regulation patterns controlled by enhancer clusters, which regulate the same gene either in a limited number of cell types (Specific pattern, Spe) or in the majority of cell types (Conserved pattern, Con), both of which are enriched for super-enhancers (SEs). We identify a previously overlooked pattern (Variable pattern, Var) that multiple enhancers link to the same gene, but rarely coexist in the same cell type. These three patterns control the genes associating with distinct biological function and exhibit unique epigenetic features. Specifically, we discover a subset of Var patterns contains Shared enhancers with stable enhancer-promoter interactions in the majority of cell types, which might contribute to maintaining gene expression by recruiting abundant CTCF.

**Conclusions:**

Together, our findings reveal three distinct E-P regulation patterns across different cell types, providing insights into deciphering the complexity of gene transcriptional regulation.

**Supplementary Information:**

The online version contains supplementary material available at 10.1186/s12864-024-10408-w.

## Background

Enhancer regulatory elements are specific non-coding DNA sequences that cis-regulate intracellular gene expression by binding with transcription factors (TFs) [1]. Multiple enhancers form cluster to regulate genes, usually associated with enhanced transcriptional activity and enrichment of key genes and super-enhancers for cell fate [2–5]. Multiple enhancers can also act in an additive, synergistic, redundant or hierarchical manner to regulate gene expression [6]. The addictive and synergistic patterns have been described at the level of SEs and exert a greater impact on controlling cell identity during development and tumorigenesis [7–9], such as SEs controlling α-globin and *MYC* genes, where gene activity increases linearly with the number of enhancers [10, 11]. Enhancer redundancy is prevalent in the mammalian genome, where multiple enhancers can buffer the risk of lethality due to the loss of individual enhancers [12, 13]. Moreover, multiple enhancers can form a hierarchical structure where those certain enhancers play a crucial role in chromatin organization and gene activation [14, 15]. Meanwhile, studies have shown that the expression of the same gene may rely on distinct enhancers in different cell types, exemplified by as *Tet2* [16] and *MYC* [10]. These studies highlight the complexity of E-P regulations. However, most of these studies have focused on investigating enhancers within individual cell type, it remains largely unexplored how multiple enhancers co-regulate gene expression across various cell types that do not overlap in space at genome scale [17–19].

Chromosome conformation capture techniques unveil the three-dimensional spatial folding of the genome, thereby facilitating long-range chromatin interactions between enhancers and promoters, leading to more complex gene regulation [20–27]. Technologies such as Hi-C [28] and its derivatives, including ChIA-PET [29], have made significant advancements in studying chromatin conformation and interactions. However, these techniques still faces the challenges such as high cost and low resolution. To overcome these challenges, algorithms utilizing DNA sequences and chromatin features have emerged. For example, the Activity-by-Contact (ABC) model predicts genome-wide E-P interactions in various cells and tissues [30–32]. These computational approaches and the available data enhance our ability to explore how multiple enhancers coordinate gene expression across various cell types at the genome scale.

In this study, we have developed an approach to systematically analyze the regulatory networks between enhancers and promoters across different cell types at the genome scale based on the E-P interactions predicted by the ABC model [30]. Specifically, we have quantified enhancer specificity and selectivity across various cell types and identified three patterns of E-P regulation: Spe, Var and Con, where Var pattern is ignored previously. Each pattern exhibits distinct characteristics in terms of chromatin accessibility, TFs binding, target gene expression and function enrichment. Overall, our findings reveal the existence of distinct E-P regulation patterns that explain the coordination of gene expression by multiple enhancers across various cell types.

## Results

### Enhancer specificity and selectivity across diverse cell types delineates three distinct patterns of E-P regulation

**Fig. 1:**
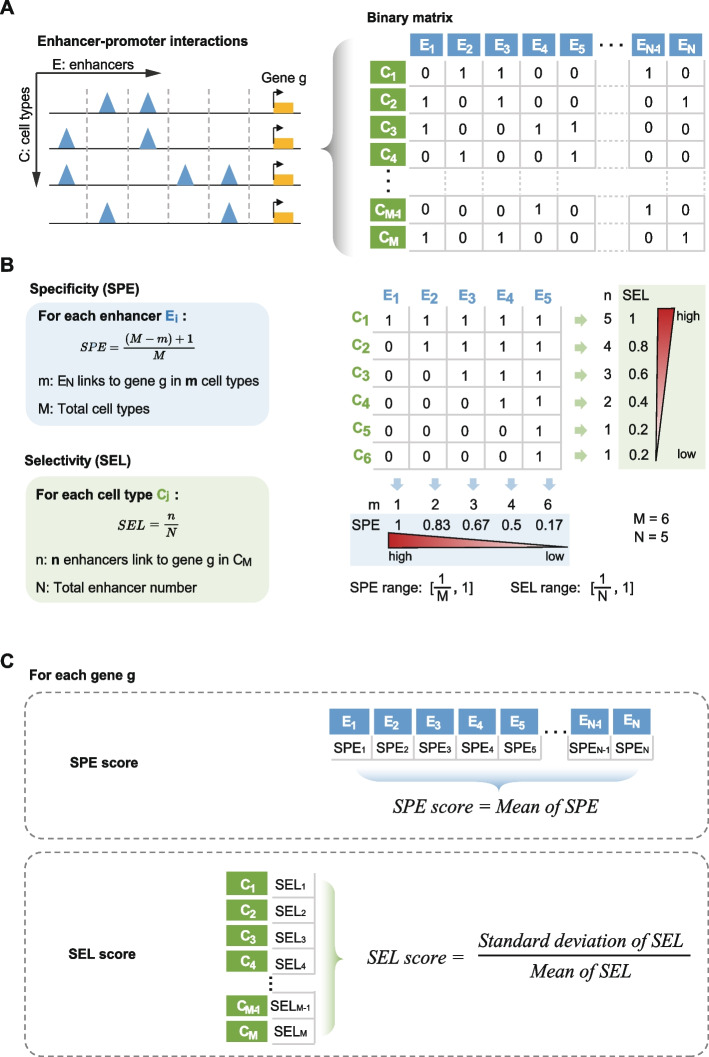
A computational approach to quantify enhancer specificity and selectivity across cell types based on E-P interactions. **A** Step1: E-P interactions as the input. For each gene, the E-P interactions across various cell types were represented by a binary matrix, where the value 1 and 0 indicated the presence or absence of an E-P interaction in individual cell type. **B** Step2: Quantification of enhancer specificity (SPE) and enhancer selectivity (SEL) across diverse cell types. The cell-type specificity for each enhancer was measured by its relative frequency across all cell types, while enhancer selectivity in each cell type was determined by the percentage of the constituent enhancers involved in interactions in the cell type. The right panel provides an example to illustrate the calculation of SPE and SEL. **C** Step3: Calculation of the SPE score and SEL score for each gene. The SPE score for a gene was computed as the mean of the specificity of its associated enhancers. The SEL score across cell types was determined by the relative standard deviation of enhancer selectivity across all cell types

In order to gain insight into how multiple enhancers co-regulate genes across diverse cell types, we developed a computational approach that quantifies enhancer specificity and selectivity based on E-P interactions data. First, for each gene, we complied a set of enhancers that exhibited E-P interactions with its promoter in at least one cell type, representing by a binary matrix (Fig. 1A). Second, we defined two metrics for each gene to delineate its E-P regulation patterns across various cell types (Fig. 1B). Given a gene, enhancer specificity (SPE) indicates the cell specificity of each enhancer, while enhancer selectivity (SEL) represents the proportion of enhancers selected to control its expression in each cell type. SEL is quantified by the number of enhancers linked to the gene in a specific cell type divided by the total number of enhancers linked across various cell types (Fig. 1B). The SPE score quantifies the overall specificity of enhancers associated with a gene across different cell types, calculated as the average SPE value of each enhancer. A higher SPE score indicate that the gene tends to be regulated by more cell type-specific enhancers. The SEL score reflects the variability in the number of enhancers linked to a gene and is determined by the relative standard deviation of SEL in each cell type. A higher SEL score suggests that the gene is more likely to be regulated by enhancers present in a minority of cell types (Fig. 1C).

**Fig. 2:**
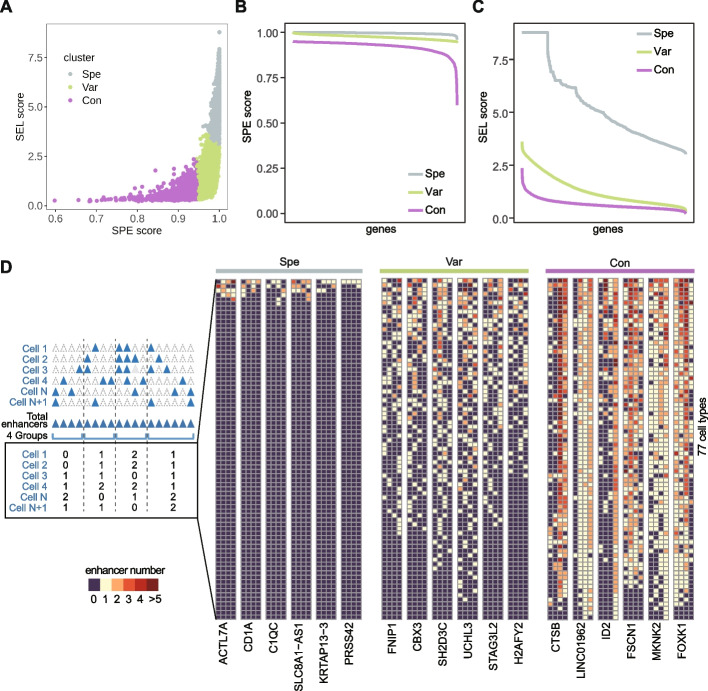
SPE score and SEL score across cell types delineates three E-P regulation patterns. **A** The SPE score and SEL score for all genes based on E-P interactions in 77 cell types (defined by ABC-model) were subjected to K-means clustering. The clustering resulted in three E-P regulation patterns: Spe, Var and Con. **B** Distribution of SPE score at genome scale in three patterns. The *x*-axis represents genes ranked by SPE score. **C** Distribution of SEL score at genome scale in three patterns. The *x*-axis represents genes ranked by SEL score. **D** Six genes representing for each three patterns were randomly selected. For each gene, the associated enhancers determined by E-P interactions were divided into four groups (column). The color indicated the number of enhancers located in each group within individual cell type. The order of cell type was determined by the total number of enhancers in each cell type

We applied our pipeline to investigate the E-P regulations across various human cell types, using publicly available E-P interactions defined by the ABC-model [30]. The dataset comprised 819,654 E-P interactions involving 22,921 genes and 229,038 enhancers across 77 cell types (Additional file 1: Figures S1A, B; see Methods). To assess the biological significance of the predicted E-P interactions by the ABC model, we collected Hi-C [33] and Hi-TrAC [34] data from six cell types. The results indicated that ABC links across all six cell types were significantly enriched for Hi-C chromatin interactions compared to the genomic control group at various E-P distances. We observed a strong correlation between the ratio of validated ABC links and the resolution of proximity ligation-based chromatin interaction data. Specifically, Hi-TrAC data for K562 and GM12878 with higher resolutions, supported over 40% of ABC links, while the remaining four datasets with resolutions approximately ranging from 5k to 10k showed slightly lower validation rates. Overall, the E-P interactions predicted by the ABC model demonstrate biologically meaningful (Additional file 1: Figure S 2). To focus on the regulation of multiple enhancers, we excluded genes associated with only one enhancer (135 genes) from further analysis. This resulted in a median of 33 enhancers per gene (Additional file 1: Figure S1C). Then, we employed K-means clustering (k=3) based on SPE score and SEL score to categorize the genes into three distinct regulation patterns: Spe, Var and Con (Fig. 2A and Additional file 1: Figures S3 and S4; see Methods).

To systematically characterize these three E-P regulation patterns across various cell types at the genome scale, we performed the following analysis. First, we ranked all genes according to their SPE score and SEL score (Figs. 2B, C). As expected, genes belonging to different patterns showed distinct distributions of SPE score and SEL score across all genes. Furthermore, we randomly selected some genes from each pattern to visualize the distribution of their associated enhancers. Considering that the number of associated enhancers varies among genes and cell types, we divided the all enhancers for each gene equally into four groups and counted the enhancers with E-P interactions in each regions specific to a given cell type (Fig. 2D, left panel). The results showed that these E-P interactions exhibit three completely different distribution patterns (Fig. 2D, right panel). As mentioned earlier, a higher SPE score indicated the gene is likely regulated by enhancers specific to certain cells, as depicted in the heatmap where Spe and Var enhancers were limited in a minority of cell types compared to Con. Conversely, a lower SEL score indicated the gene is connected to enhancers across various cell types, as shown in the heatmap where Var and Con genes linked a certain number of enhancers in multiple cell types (Fig. 2D). This finding confirmed the effectiveness of SPE scores and SEL scores in classifying E-P regulatory relationships. In conclusion, we define the SPE score and SEL score as metrics to classify the enhancer-gene regulatory relationships across various cell types into three patterns. Spe represents a cell-specific regulation pattern, while Var represents a widespread but cell-dependent regulation pattern, and Con represents a widely present and conserved regulation pattern.

### Three regulation patterns control the expression of functionally distinct genes

**Fig. 3:**
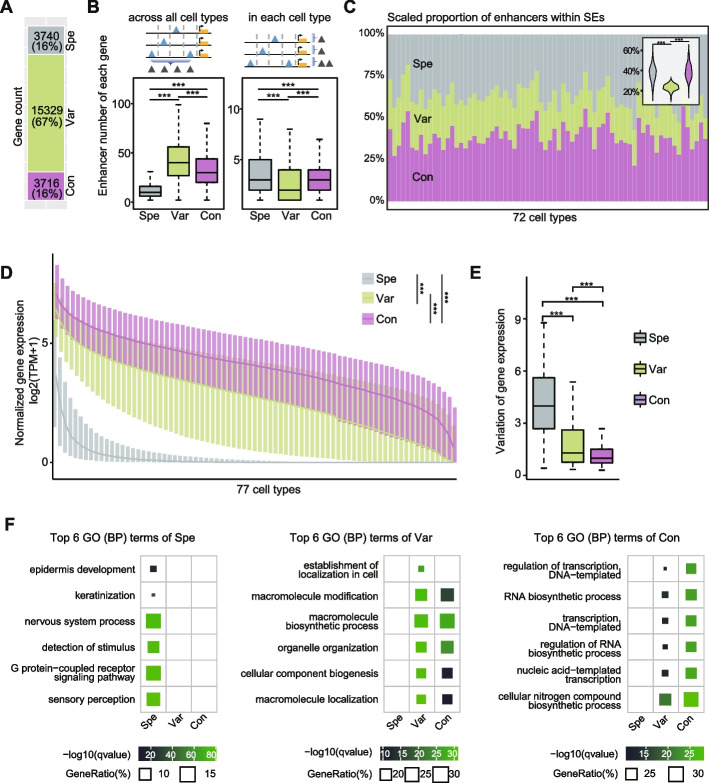
Three regulation patterns control genes with distinct biological functions. **A** The number of genes controlled by three patterns. **B** The number of enhancers linked to each gene across all cell types and in a single cell type. The top panel illustrates the statistical analysis of enhancers. **C** Percentage of enhancers in three patterns located within super-enhancer regions was shown for each cell type. The *y*-axis indicates the percentage of three patterns scaled to 100%. **D** The expression levels of genes in three regulation patterns. The *x*-axis indicates the cell type order for each gene ranked by its expression value. **E** Variation in gene expression across cell types for genes. Variation was represented by the relative standard deviation. **F** GO enrichment analysis. P-values were calculated using Wilcoxon test. *p < 0.05; **p < 0.01; ***p < 0.001; *n.s.*, not significant

Here, we identified a total of 3,740, 15,329 and 3,716 genes belonging to Spe, Var and Con patterns, respectively (Fig. 3A). We first compared the number of enhancers associated with genes and found that Var genes were associated with a significantly higher number of enhancers across all cell types but fewer enhancers within individual cell type compared to Spe and Con genes (Fig. 3B). This implied that Var genes in each cell type were regulated by the fewest enhancers, yet these highly cell-specific enhancers were broadly distributed across various cell types. SEs have been known to control expression of genes that define cell identity [3]. It is worth noting that Var enhancers showed the lowest proportion of SEs in different cells (Fig. 3C; see Methods), distinguishing them from the known SE regulation pattern.

Furthermore, genes controlled by these three patterns displayed distinct expression trends. Spe gene expression exhibited high cell specificity and the lowest overall level. In contrast, Var and Con genes were expressed in most of cell types, with Con genes exhibiting higher expression overall compared to Var genes (Figs. 3D, E). GO analysis revealed that Spe genes were associated with various cell-specific functions, such as sensory perception of neural cells and keratinization of epithelial cells (Fig. 3F). This finding aligns with the notion that cell-specific enhancer clusters playing a critical role in determining cell identity [3]. Interestingly, although both Var and Con genes expressed in multiple cell types, they were enriched for distinct biological functions (Fig. 3F). Var genes were mainly involved in the synthesis of macromolecules and organelle assembly, while Con genes were predominantly associated with in DNA, RNA and gene expression-related functions that are fundamental to cellular life (Fig. 3F). Additionally, a significant proportion of housekeeping genes fell into the Var and Con categories, with Con genes having the highest proportion of housekeeping genes [35] (Additional file 1: Figure S5). This also demonstrated that Var and Con genes were involved in more fundamental and conserved functional modules. Taken together, these findings demonstrate that the three regulation patterns identified in our study control functionally distinct genes, exhibiting specific gene expression patterns across diverse cell types.

### The three regulation patterns display distinct epigenetic features

**Fig. 4:**
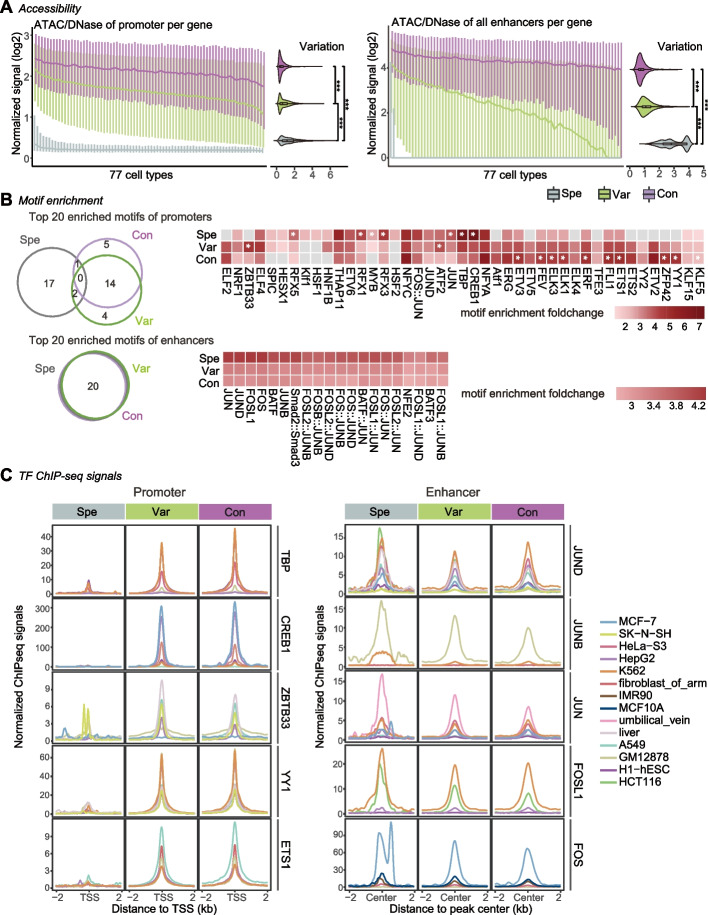
The epigenetic characteristics of enhancers and promoters in the three E-P regulation patterns. **A** Chromatin accessibility of the promoter (left) and enhancer (right), measured using DNase-seq or ATAC-seq (if ATAC-seq data is unavailable). The enhancer accessibility of each gene was the sum of all enhancer accessibility. The cell types were ordered consistently with **Fig. 3D**. **B** Motif enrichment analysis. The Venn diagrams illustrated the number of shared motifs among the top 20 enriched motifs in promoters (left) and enhancers (right), ranked by q-values. The heatmap showed the fold-enrichment of the top20 motifs using random genome sequences as background. The asterisk indicated that the motifs showed more than 1.5-fold enrichment in this pattern compared to the lowest pattern. **C** The signal intensity plots of TF ChIP-seq for promoters (left) and enhancers (right). The *x*-axis represents the region of TSS ±2kb (left) and the region of enhancer center ±2kb (right)

To further characterize the epigenetic features and understand the underlying mechanisms in three regulation patterns, we next compared the chromatin accessibility of enhancers and promoters. We found that the Con pattern showed the highest accessibility in both promoters and enhancer across all cell types. This was followed by the Var pattern, while the Spe pattern displayed the lowest accessibility (Fig. 4A). These results aligned with the observations we made in gene expression (Fig. 3D). Furthermore, the higher correlation between gene expression and the promoter accessibility also suggested that the explanatory capacity of promoters exceeds that of enhancers in elucidating the regulation of gene expression (Additional file 1: Figure S6). The accessibility of the three types of promoters remained stable across cell types compared to enhancers, which exhibited significantly different variations (Fig. 4A). This suggested that enhancers play a dominant role in determining the cell-specificity level of gene expression.

Interestingly, motif enrichment analysis revealed that the top 20 enriched motifs in promoters of three patterns were divergent, while the motifs of enhancers were almost identical (AP-1 family) (Fig. 4B). To investigate this further, we selected some TFs with high motif enrichment of different patterns from the top 20 motifs and obtained the corresponding ChIP-seq data (Additional file 2: Table S 1). The results showed that at the promoter region, all TFs were more enriched in Var and Con than those in Spe. Even TBP and CREB1, which had higher motif enrichment in Spe, showed the lowest signal intensity (Fig. 4C, left panel and Additional file 1: Figure S7). In contrast, the signal strength of TFs of Spe enhancers is even slightly stronger than the other patterns (Fig. 4C, right). These results suggested the TF composition varies across promoters, reflecting diverse transcriptional environments for genes with accessible promoters. Meanwhile, Var and Con have higher TF intensity than Spe, which is the primary factor contributing to the substantial difference in expression among these gene types. Conversely, the enhancers showed an opposite pattern, indicating that regardless of accessibility, the enhancers in three patterns maintain comparable TF strengths. Notably, genes with weak promoter accessibility, such as Spe, required more enhancers to sustain TF intensity and activate gene expression (Figs. 3B and 4C).

Taken together, our results highlight distinct features of the three patterns in terms of promoters and enhancers, including chromatin accessibility, motif enrichment, TF binding, and their correlation with gene expression (Additional file 1: Figure S8).

### A subset of Var pattern contains Shared enhancers with stable E-P interactions across the majority of cell types

**Fig. 5:**
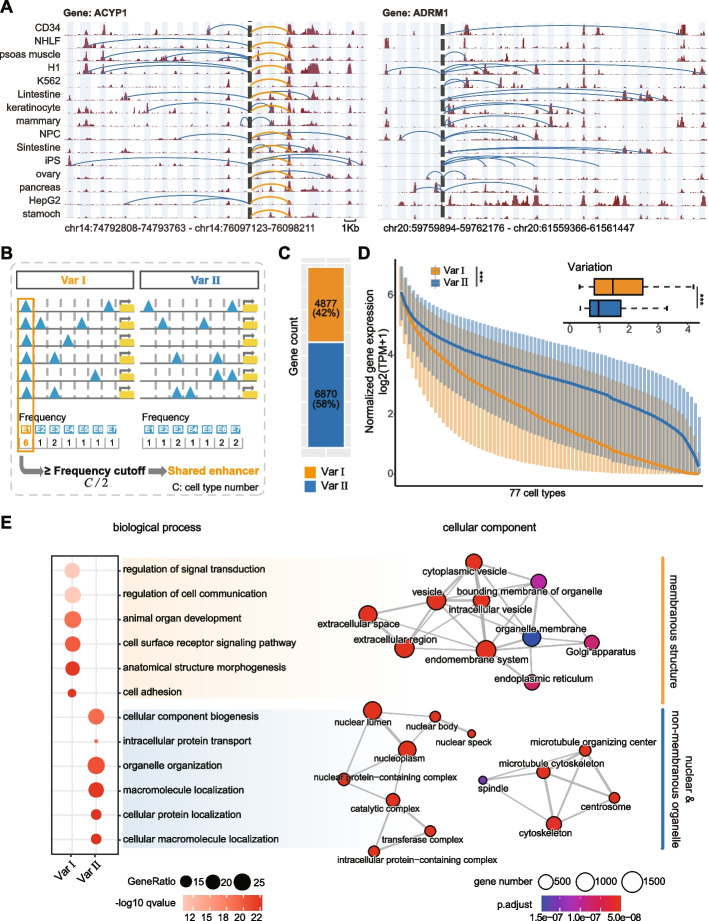
Var regulation pattern contains two distinct E-P regulation subtypes, Var I & Var II. **A** Examples of Var I (left) and Var II (right) regulation subtypes. The gray dashed line denoted the promoter region, while the light blue rectangles represented enhancer regions. The arcs denoted E-P regulation relationships defined by ABC model, with blue arcs representing high cell-specific E-P interactions and yellow arcs representing E-P interactions present in the majority of cell types. For demonstration purposes, only part of the cell line was shown and the enhancers were stitched together and do not represent real distances. The genomic loci of the first and last enhancer were indicated at the bottom. **B** Definition of Shared enhancers, which divided Var regulation pattern into two distinct E-P organization subtypes, Var I and Var II. **C** The number of genes controlled by Var I and Var II regulation subtypes. 3,582 Var genes with RPKM less than 1 or lacking RNA-seq data in all cell types were excluded from the analysis. **D** Expression levels of Var I and Var II genes. The *x*-axis indicates the cell type order for each gene ranked by its expression value. Variation was represented by the relative standard deviation. **E** GO enrichment analysis. P-values were calculated using Wilcoxon test. *p < 0.05; **p < 0.01; ***p < 0.001; *n.s.*, not significant

Var exhibited a distinct E-P regulation pattern where each gene was associated with multiple enhancers, but these enhancers rarely coexisted in the same cell type. While previous studies have focused on the phenomenon of multiple enhancers controlled the same gene in the same cell type, the coordination and impact on gene expression of enhancers in Var across various cell types, remained largely unexplored. To this end, we further investigated the distribution of E-P interactions in Var. Interestingly, we observed a subset of Var genes showed a unique regulation pattern. In this pattern, a certain enhancer consistently interacted with the same gene across most of cell types, while the remaining enhancers formed cell-type-specific interactions. For example, the 14^th^ enhancer of the *ACYP1* gene maintained a regulatory relationship with the *ACYP1* promoter in almost all cell types (Fig. 5A, left panel). In contrast, other genes in Var e.g. the *ADRM1* gene, exhibited only cell-type specific E-P interactions (Fig. 5A, right panel).

To further understand the function and mechanisms underlying the two regulation patterns, we classified enhancers that exhibited E-P interaction with the same gene in more than half of the cell types as ‘Shared’ enhancers. Subsequently, we systematically subdivided Var genes into two categories: Var I, which included genes with Shared enhancer, and Var II, which consisted of genes without Shared enhancers (Fig. 5B; see Methods). This classification resulted in 4,877 in Var I and 6,870 genes Var II (Fig. 5C). Genes in Var I showed lower overall expression levels across various cell types compared to those in Var II. However, they exhibited higher expression variations across cell types (Fig. 5D). Strikingly, GO analysis revealed distinct functional association for Var I and Var II regulated genes. Specifically, Var I genes were significantly enriched in membrane-specific structures such as membrane organelles, particularly vesicles (Fig. 5E, left panel). On the other hand, Var II genes were enriched in non-membrane organelles and internal structures of the nucleus. (Fig. 5E, right panel). In summary, Var I gene expression exhibited greater variability and was primarily associated with the dynamic membrane network. In contrast, Var II gene expression was higher and relatively stable, and these genes played a role in the assembly of other organelles.

Collectively, our findings identify a group of Shared enhancers, which maintain consistent E-P interactions across most cell types, and refine two distinct E-P organizations, which control genes associated with membrane-related and non-membrane cellular functions.

### Shared enhancers maintain gene expression by recruiting abundant CTCF and are functionally important

**Fig. 6:**
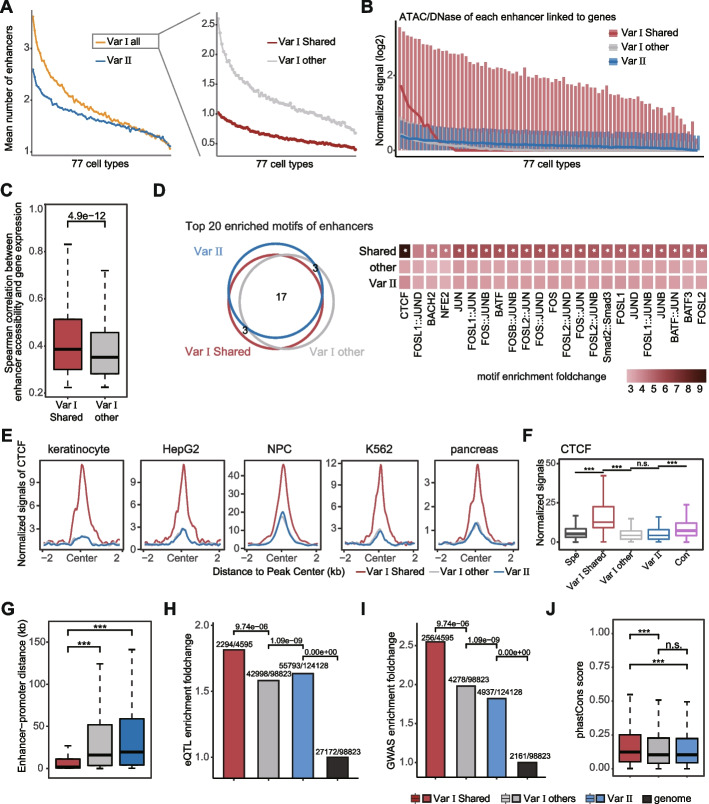
Epigenetic landscape of promoters and enhancers in Var I and Var II patterns. **A** The average number of different enhancers for Var I and Var II genes. **B** The chromatin accessibility of different enhancers in 77 cell types, measured using DNase-seq or ATAC-seq (if ATAC-seq data is unavailable). **C** The correlation between gene expression and enhancer accessibility. Spearman correlation value were calculated for each gene in 77 cell types, and the values with q-value greater than 0.05 were excluded. **D** Motif enrichment analysis. The Veen plot and heatmap showed the overlap and fold-enrichment of the top 20 enriched motifs ranked by q-value in Var enhancers, respectively. The asterisk indicated that the motifs showed more than 1.5-fold enriched in this class of enhancers than the lowest class. **E** The CTCF signal intensity within ±2kb of different Var enhancer centers in various cell types. **F** The signal strength of CTCF within ±25bp of the enhancer center in all regulatory patterns. **G** The E-P distance for different Var enhancers. The E-P distance was defined as the distance from the enhancer center to the TSS. **H**,** I** Enrichment of eQTL SNPs **H** and GWAS SNPs **I** for different Var enhancers, using randomly selected genomic regions as the control. The first number of each label represented the number of enhancers that overlapped with eQTL/GWAS SNPs, and the second number represented the total number of enhancers. The group “Genome” represented genome randomly selected sequences. **J** PhastCons conservation scores for different Var enhancers. The *y*-axis is the mean of the conservation scores of all base pairs per enhancer. The *x*-axis order in **A**,** B** is consistent with **Fig. 5D**. P-values in **C, G, J** were calculated using Wilcoxon test. *P*-values in **H**,** I** were calculated using the binomial test. *p < 0.05; **p < 0.01; ***p < 0.001; *n.s.*, not significant

We further investigated the characteristics and chromatin landscape of Var I and Var II patterns. Strikingly, Var I genes were associated with a higher number of enhancers across various cell types than those in Var II (Fig. 6A, left panel). However, both promoters and enhancers of Var II exhibited significantly higher chromatin accessibility than those of Var I (Additional file 1: Figure S9), which was consistent with their respective gene expression trends (Fig. 5D). This indicated chromatin accessibility, rather than number of enhancers, plays an important role in determining gene expression in Var regulation patterns.

We then analyzed the different enhancers in Var I separately and found that the variation in enhancer number was primarily derived by cell-specific enhancers, while the number of Shared enhancers remained relatively stable, averaging around one per cell type (Fig. 6A, right panel). Significantly, Shared enhancers consistently exhibited superior accessibility compared to other cell type-specific enhancers and demonstrated a stronger correlation with gene expression (Figs. 6B, C). Furthermore, genes with Shared enhancers in the regulation network exhibited significantly higher expression levels than genes exclusively regulated by cell-specific enhancers (Additional file 1: Figure S10). This suggested that Shared enhancers have a greater capacity to activate genes and stably regulate genes across most cell types. Motif analysis revealed that promoters with different accessibility of Var genes have a preference for motifs, with Var I being more enriched for CREB1 and TBP, and Var II being more enriched for the ETS family, which was consistent with the previous results. However, overall, the intensity of TF signaling on the Var II promoter was slightly higher (Fig. 4B and Additional file 1: Figure S11). The Shared enhancers in Var I patterns were significantly enriched in CTCF motifs and AP-1 family compared to other enhancers (Fig. 6D). This finding was further supported by CTCF ChIP-seq data, which confirmed the strong signal of CTCF binding on the Shared enhancers in various cell types. However, the enrichment of AP-1 exhibited no significant difference (Fig. 6E and Additional file 1: Figure S12). We also performed CTCF analysis for enhancers in other regulatory patterns to investigate whether similar CTCF enrichment exists. The results showed that Var I Shared enhancers exhibited the highest enrichment of CTCF binding compared to other types of enhancers (Fig. 6F and Additional file 1: Figure S12B). Moreover, the E-P regulatory distance of Shared enhancers, with a median of 18 kb, was significantly shorter than that of other enhancers (Fig. 6G). This suggested that Shared enhancers have a preference for positioning themselves around Var I genes and maintaining interactions with the gene by recruiting abundant CTCF in various cell types, thereby regulating gene expression.

To assess the impact of different Var enhancers in gene expression and disease development, we utilized single-nucleotide polymorphisms (SNPs) associated with diverse phenotypic traits and diseases from genome-wide association study (GWAS) [36] and expression quantitative trait loci (eQTLs) data. The results showed that both eQTLs and GWAS exhibited the highest enrichment in Shared enhancers (Figs. 6H, I). Additionally, we calculated phastCons scores [37] to represent constraint levels of different enhancers, supporting the enrichment of SNPs. Consistently, the conserved scores of Shared enhancers were significantly higher than those of other enhancers (Fig. 6J). This indicated that Shared enhancers were functionally more important in diseases.

Taken together, these findings suggest that Shared enhancers regulate the expression of Var I genes in different cells by recruiting CTCF to maintain E-P interactions and they are functionally important.

## Discussion

**Fig. 7:**
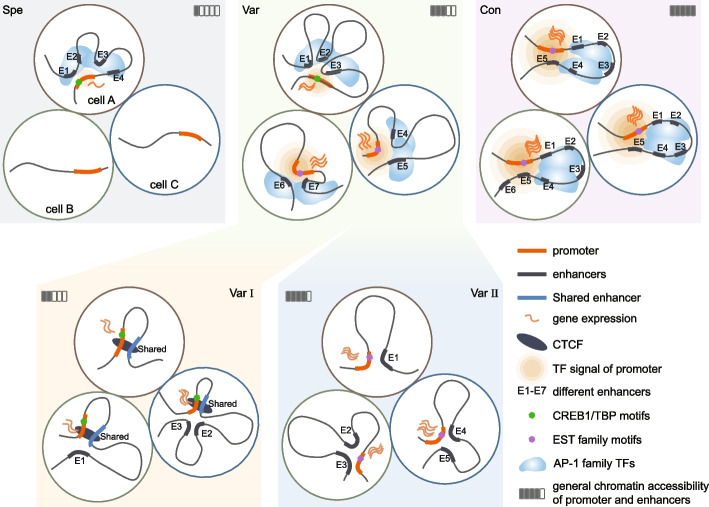
Schematic diagram illustrating the three E-P regulation patterns across different cell types. Spe pattern is characterized by specific E-P interaction limited to individual cell types, showing the lowest gene expression, chromatin accessibility and TF signal intensity. Con is distinguished by highly conserved E-P interactions across different cell types, exhibiting the highest levels of gene expression, chromatin accessibility, and TF signal intensity across all cell types. Var represents a previously overlooked pattern where the same gene is regulated by distinct enhancers across different cell types. Var is further divided into Var I and Var II based on the presence of Shared enhancers. Shared enhancers established stable interactions with target genes by recruiting abundant CTCF in most cell types, while other cell-specific enhancers amplified the expression. Although Var II lacks Shared enhancer, it maintains stable high gene expression due to high accessibility of both the promoter and enhancers. Three different types of enhancers showed similar enrichment of AP-1 motifs, while the promoters show distinct preferences. Specifically, the weakly accessible promoters (Spe) prefer binding with TBP and CREB1, while strongly accessible promoters (Var and Con) prefer binding with the EST family

Multiple enhancers coordinating the same gene expression across various cell types is prevalent in genome; however the underlying mechnism remains largely unexplored. In this study, we developed a computational approach to categorize the extensive E-P interactions across 77 different cell types into three distinct patterns: Specific pattern (Spe), Variable pattern (Var) and Conserved pattern (Con). These patterns exhibited diverse regulatory characteristics, including gene function (cell-specific to conserved), enhancer and promoter accessibility (low to high), and gene expression variability (variable to stable) (Fig. 7). While a prior study identified active genomic regions with stronger transcriptional activity based on transcription factor binding affinity and Hi-C data [38], it remained unclear how regulatory elements such as enhancers control genes with varying transcriptional activity across cell types. Our focus on enhancer-gene pairs and the utilization of enhancer specificity and selectivity across a broad range of cell types allowed us to elucidate how the same genes were precisely regulated across various cell lineages.

Spe and Con represented two opposite regulatory patterns. The E-P interactions of Spe genes were confined to individual cell types, indicating strong cell specificity, while the E-P links of Con gene were universally present in most cell types, demonstrating pronounced conservatism. Additionally, SPE and Con also exhibit different preferences in various epigenetic features. Weakly accessible Spe promoters displayed a preference for the CPB/CREB1 motif, whereas highly accessible Var and Con promoters favored the EST/YY1 motif. This suggested motif preferences at promoters with different accessibility levels [39]. However, unlike promoters, weak Spe enhancers displayed comparable AP-1 enrichment strength to Var and Con enhancers (Figs. 4B, C and Additional file 1: Figure S8). This finding aligned with a study conducted in Drosophila that demonstrated higher concentrations of TFs are required for activation of weakly active *hb* gene promoter transcription [40]. The weak promoter of cell-specific functional genes potentially serves as a protective mechanism against transcriptional abnormalities [41]. In contrast, densely conserved enhancers in the Con pattern recruit elevated concentrations of transcription factors, thus ensuring robust gene expression [42].

Furthermore, Var represented a unique regulatory pattern where the same gene is controlled by multiple enhancers without co-existing in the same cell type. This suggested that Var harbors the largest collection of cell-specific enhancers, despite the limited number within each cell type. Var was further divided into Var I and Var II based on the presence of Shared enhancers. Shared enhancers establish stable interactions with target genes (Var I genes) in most cells by recruiting abundant CTCF, maintaining the basic expression of genes (Figs. 6E, F). The distance of Shared E-P link was approximately 20kb, suggesting that Shared enhancers possess a spatial advantage over other enhancers, prioritizing their connection to genes (Fig. 6G). Other cell-specific enhancers contributed to the variability of gene expression across different cell types. Although the enhancers of Var II exhibited similar epigenetic characteristics to Var I other enhancers, Var II genes maintained a more stable and widespread expression through highly accessible promoters (Fig. 5D and Additional file 1: Figure S9). This suggested that transcriptional activation of variably expressed genes was more dependent on distal enhancers, while widely expressed genes were relatively insensitive to distal enhancers [41]. Overall, Var pattern might represent a less costly regulatory mechanism in the genome, utilizing a minimum number of enhancers to control the expression and variation of ubiquitously expressed genes.

Although we have deciphered three regulatory patterns of E-P interaction, the impact of interactions between TF-enhancer, TF-TF on genes remained unclear. A recent study revealed that pioneer factors facilitated CTCF occupancy to control the expression of cell identity genes [43]. Based on the results of CTCF enrichment and variable accessibility of Var enhancers, we wondered if cell type-specific pioneer factors also facilitate the collaboration between other enhancers and Shared enhancers and affect their accessibility. This intriguing point requires exploration with additional data in future studies.

## Conclusion

Here, we develop a computational approach that enables the quantitative assessment of enhancer specificity and selectivity across diverse cell types. Our findings identify three distinct regulation patterns of E-P interactions across various cell types. These patterns highlight the heterogeneity of enhancer regulatory mechanisms of genes with different functional modules, which provides new insights to the subtle mechanisms of enhancers in transcriptional regulation.

## Methods

### Preparation of the E-P interactions in 77 human cell types

In this study, we selected 77 cell types out of 131 cell types and tissues from ABC Model predictions [30]. We retained cell types with availability of various omics profiles and removed repetitive cell types. E-P interactions for these 77 cell types were extracted from ABC Model predictions. We specifically focused on E-P links with an ABC score greater than 0.015, which were defined as predicted E-P interactions [30]. The genomic coordinates of the peaks and target gene information were extracted from the dataset, with no further consideration of the ABC score. The dataset used in this study can be accessed at https://mitra.stanford.edu/engreitz/oak/public/Nasser2021/AllPredictions.AvgHiC.ABC0.015.minus150.ForABCPaperV3.txt.gz. Detailed statistics of E-P interactions, as well as data sources for all cell types, can be found in Additional file 2: Table S1 and Additional file 1: Figure S1.

### Compilation of enhancer coordinates

To ensure comparability of enhancers across different cell types, we unified their chromatin coordinates. First, we gathered the peak coordinates from all cell types into a single file. Then, using bedtools [44] merge function with default parameters, we merged the overlapping peaks, generating a peak set with uniform and non-overlapping coordinates. Next, we utilized bedtools intersect function to overlap the original enhancers from each cell type with the unified peak set. The enhancers that overlapped with unified peaks were replaced by the unified coordinates. In this study, we defined the regions within +/-1kb of any RefSeq annotated transcription start site (TSS) as promoters, while peaks outside of the promoter regions were considered as enhances.

### Calculation of SPE score and SEL score

For each gene (g), we generated a binary matrix of size M (rows) * N (columns) to represent the E-P interactions across various cell types. M represents the number of cell types (M = 77 in this study), while N represents the total number of enhancers that regulate the gene in all cell types based on the E-P interactions defined by ABC model [30]. Each element *x*_*ij*_ in the matrix represents the value at the *i*_*th*_ row (cell type) and *j*_*th*_ column (enhancer):

- *x*_*ij*_ = 0, the enhancer does not link to gene g in this cell type;
- *x*_*ij*_ = 1, the enhancer links to gene g in this cell type.

#### SPE score

We calculated the Enhancer Specificity (SPE) for each enhancer e using the following formula:

$$\text{SPE = }\frac{\left({\text{M}}-{\text{m}}\right)\text{+}{1}}{\text{M}}$$

Here, *m* represents the number of cell types in which the enhancer e regulates the gene g. For the gene g, we calculated the SPE score, which is the average of the SPE values for all enhancers associated with gene g:

$$\text{SPE score = }\text{Mean of SPE}$$

#### SEL score

We calculated Enhancer Selectivity (SEL) for each cell type (c) using the following formula:

$$\text{SEL = }{\text{n}}\text{/}{\text{N}}$$

Here, *n* represents the number of enhancers that regulates the gene g in cell type c and *N* represents the total number of enhancers associated with gene g across all cell type. For the gene g, we calculated the SEL score, which is the relative standard deviation of SEL values across all cell types:

$$\text{SEL score}\text{ = }\frac{\text{Standard deviation of SEL}}{\text{Mean of SEL}}$$

These sores, the SPE score and SEL score, provided quantitative measures to delineate the enhancer specificity and selectivity of gene g across various cell types, respectively.

### Classification of E-P regulation patterns based on SPE score and SEL score

We classified genes based on their SPE score and SEL score using K-means clustering. The choice of three clusters were primarily determined based on two considerations: the clustering patterns by SPE/SEL scores (Fig. S3) and the interpretability of functional analysis (Fig. S4). When selecting k=3, the SPE/SEL scores and gene function exhibited the most distinct and non-repetitive grouping results.

### Calculation of the proportion of enhancers overlap with SEs

SEs for 72 cell types were downloaded from SEdb 2.0 [45]. The cell type IDs corresponding to these SEs can be found in Additional file 2: Table S1. We extracted the SE coordinate from the downloaded file. We then determined the the number of enhancers that are located within SE regions in each cell type using bedtools intersect function. This allowed us to assess the extent of overlap between enhancers and SE regions.

### Gene Ontology (GO) enrichment analysis

GO enrichment analysis was performed using the enrichGO function of the R package clusterProfiler [46] based on the org.Hs.eg.db database. Terms with q-values less than 0.05 were considered to be significantly enriched. The tree clustering of GO term was performed using R package enrichplot.

### Processing of ATAC-seq, DNase-seq, and ChIP-seq data

To calculate the strength of the epigenetic features for different enhancers and promoters, we collected bigwig files or fastq files of ATAC-seq, DNase-seq, and ChIP-seq data from Roadmap Epigenomics Project [47], Encode project [48], and published data or studies [30, 49, 50], respectively. The hg38 bigwig files were converted to hg19 using CrossMap [51]. All fastq libraries were checked using FastQC , while the low-quality reads and adaptor sequences were trimmed by fastp [52] and cutadapt [53]. The trimmed reads were mapped to the human reference genome (hg19) using Bowtie2 [54] with default parameters. Bam files of duplicated cell types were merged using samtools [55] merge function. Duplicated reads were removed using the MarkDuplicates function from PicardTools. Mitochondria reads were removed by samtools view function with the parameter ‘-b –L’. Genome blacklist regions [56] were excluded using 'bedtools intersect -v'. Then the bam file for each cell type was converted into bigiwig file using deepTools [57] bamCoverage with options‘–binSize 10 –normalizeUsing BPM’. The bigwig file was used to calculate the signal values for a given genomic region using the BED-file mode of the multiBigwigSummary function of deepTools [57]. Quantile normalization was applied to normalize the signal values across different cell types using the preprocessCore package. Of note, when calculating the accessibility of enhancers, all enhancers were unified to a length of 500bp (peak center +/- 250bp). All download IDs were listed in Additional file 2: Table S1.

### Processing of RNA-seq data

To calculate the gene expression, we collected raw read count files or TPM files of RNA-seq data from Roadmap Epigenomics Project [47], Encode project [48], Cancer Cell Line Encyclopedia (CCLE) [58] and published data or studies [59–61], respectively. Raw read count files were used to calculate TPM. The exon lengths of the genes were extracted using exonsBy function from the R package GenomicFeatures [62]. Quantile normalization was applied to normalize the TPM values across different cell types using the preprocessCore package. All download IDs were listed in Additional file 2: Table S1.

### Motif enrichment analysis

Motif enrichment analysis was performed using the HOMER (http://homer.ucsd.edu/homer/) findMotifsGenome.pl script. Motifs with q-values less than 0.05 were considered to be significantly enriched. To determine the fold-change of motif enrichment, we calculated the ratio between the percentage of target sequence with the motif and the percentage of background sequences with the motif.

### Classification of Var I and Var II regulation patterns

Genes with low expression levels, defined as a TPM value less or equal to 1 were excluded from further analysis. Additionally, genes lacking RNA-seq data across cell types were also discarded. For the remaining genes, we examined their E-P interactions. Enhancers that interacted with a gene in more half of the cell types were defined as Shared enhancers, while the remaining enhancers linking to this gene as other enhancers. This regulation pattern containing Shared enhancers was classified as Var I, while the regulation pattern lacking Shared enhancers was classified as Var II.

### Visualization of TF ChIP-seq signals for promoters and enhancers

The signal intensity of TF for promoters and enhancers were calculated using the computeMatrix reference-point function from deepTools with the following parameters ‘--referencePoint center -a 2000 -b 2000 --binSize 10’. The visualization was completed using an in-house R script.

### Enrichment analysis of eQTL and GWAS SNPs

The eQTL data and GWAS data were downloaded from GTEx [63] and UCSC Table Browser [64], respectively. The enhancers overlapped with GWAS SNPs were identified using bedtools intersect function. The enrichment fold-change for enhancers was calculated as follows:

$$\frac{{\text{m}}\text{/}{\text{M}}}{{\text{n}}\text{/}{\text{N}}}$$

where *m* represents the number of enhancers with SNP loci, *M* represents the number of genome random regions with SNP loci, *n* represents the total number of enhancers, and *N* represents the total number of genome random regions. To ensure comparability, all enhancers were unified to a size of 500bp (peak center +/- 250bp). The genomic random regions were 500 bp sequences randomly selected using 'bedtools shuffle' in genome-wide while excluding the promoter regions.

## PhastCons conservation score of enhancers

To determine the PhastCons conservation score for enhancers, we obtained the PhastCons 100-way vertebrate conservation score files [37] for all chromosomes of hg19 from UCSC Genome Browser [64]. We then converted the download .wig files into .bedgraph files using UCSC using wigToBigWig and bigWigToBedGraph. The PhastCons score for each enhancer was represented by the average of the scores of each base calculated using ‘bedtools intersect -a peak.bed -b PhastCons.bedgraph’. Bases without a PhastCons were excluded from the analysis.

### Supplementary Information

Additional file 1: Supplementary Figures. Figure S1 – Figure S12.

Additional file 2: Table S1. Summary of data used in this study.

## Data Availability

Predicted E-P interactions of 77 cell types were extracted from ABC Model predictions. Raw data and processed data of RNA-seq, ATAC-seq, DNase-seq, and ChIP-seq were downloaded from Roadmap Epigenomics Project, Encode project, Cancer Cell Line Encyclopedia (CCLE) and published studies GSE190792, GSE215792, GSE75384, GSE124191, GSE155555, GSE84578, respectively. SEs for 72 cell types were downloaded from SEdb 2.0. All download IDs were listed in Additional file 2: Table S1. The source code for the SPE score and SEL score can be found at this link: https://github.com/xmuhuanglab/eSelectivity.
