# Supplementary material for: Enhancer selectivity across cell types delineates three functionally distinct enhancer-promoter regulation patterns
Source: BMC Genomics. 2024 May 16;25:483. doi: 10.1186/s12864-024-10408-w (PMC11097474; doi:10.1186/s12864-024-10408-w)
Supplement: Supplementary file 1 — Additional file 1: Supplementary Figures. Figure S1 – Figure S12. [file 12864_2024_10408_MOESM1_ESM.docx]

**Fig. S1**

**
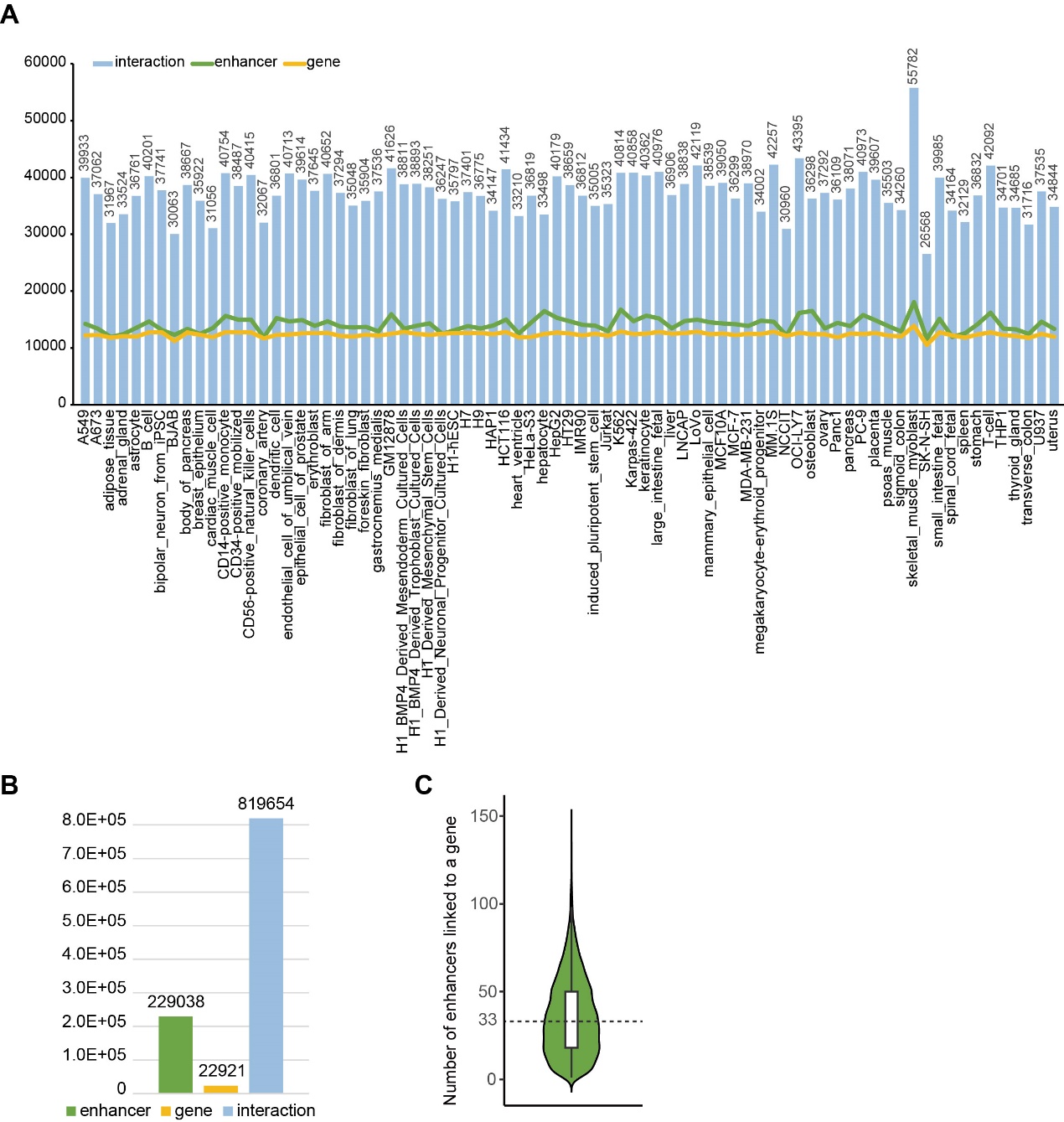
**

**Fig. S1 Enhancer-promoter interactions information across 77 cell types.** (**A**) Statistics of the number of enhancers, promoters and E-P interactions in each cell type obtained from ABC model. (**B**) Total number of enhancers, promoters and E-P interactions across all cell types after unified coordinates. (**C**) Median number of enhancers linked to each gene across all cell types.

**Fig. S2**

**
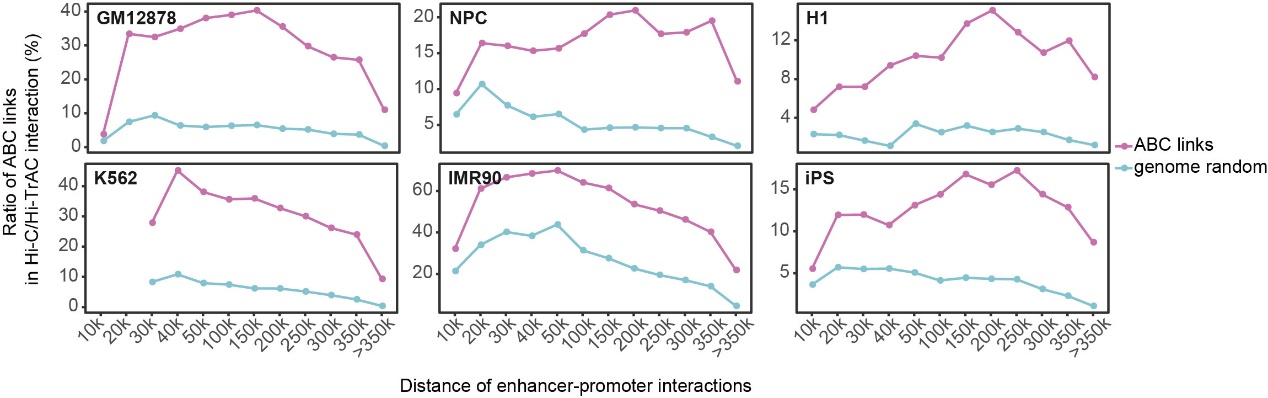
**

**Fig. S2 Ratio of ABC links detected by proximity ligation-based chromatin interaction data.** The *x*-axis indicates the ABC links were grouped based on their E-P distances. Random E-P pairs were selected within the genome as controls. GM12878 and K562 are Hi-TrAC datasets and the rest are Hi-C datasets.

**Fig. S3**

**
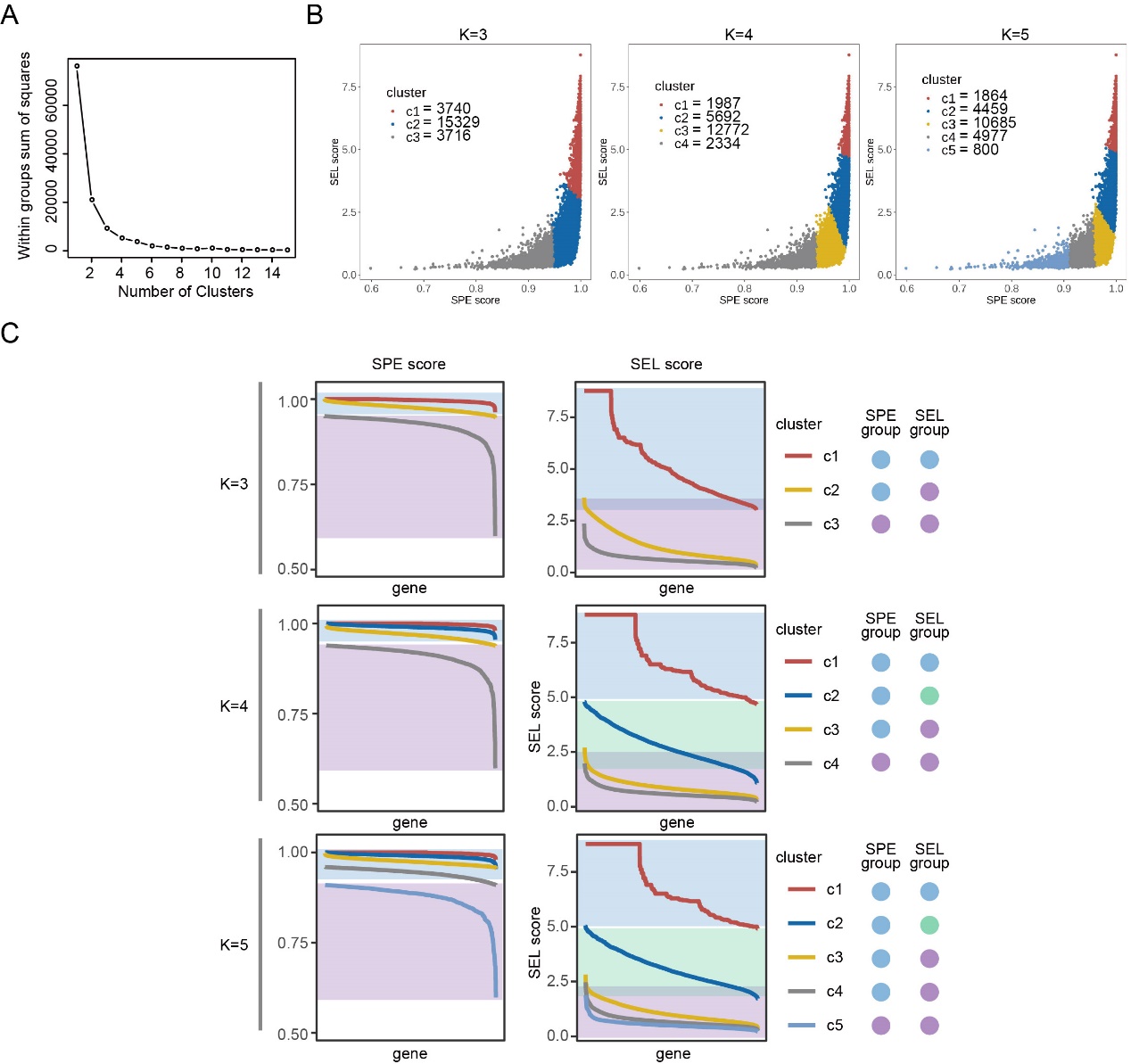
** **Fig. S3 The SPE score and SEL score of clusters classified at different k values.**

(**A**) Evaluating the optimal k value for K-means clustering using SSE (sum of the squared errors). (**B**) Results of K-means clustering for SPE score and SPE score at different k values. The numbers showed the number of genes in each cluster. (**C**) Distribution of SPE score and SEL score at genome scale of clusters classified at different k values. Circles indicated combinations of groups of the SPE score distribution and the groups of SEL score distribution at different k values.

**Fig. S4**

**
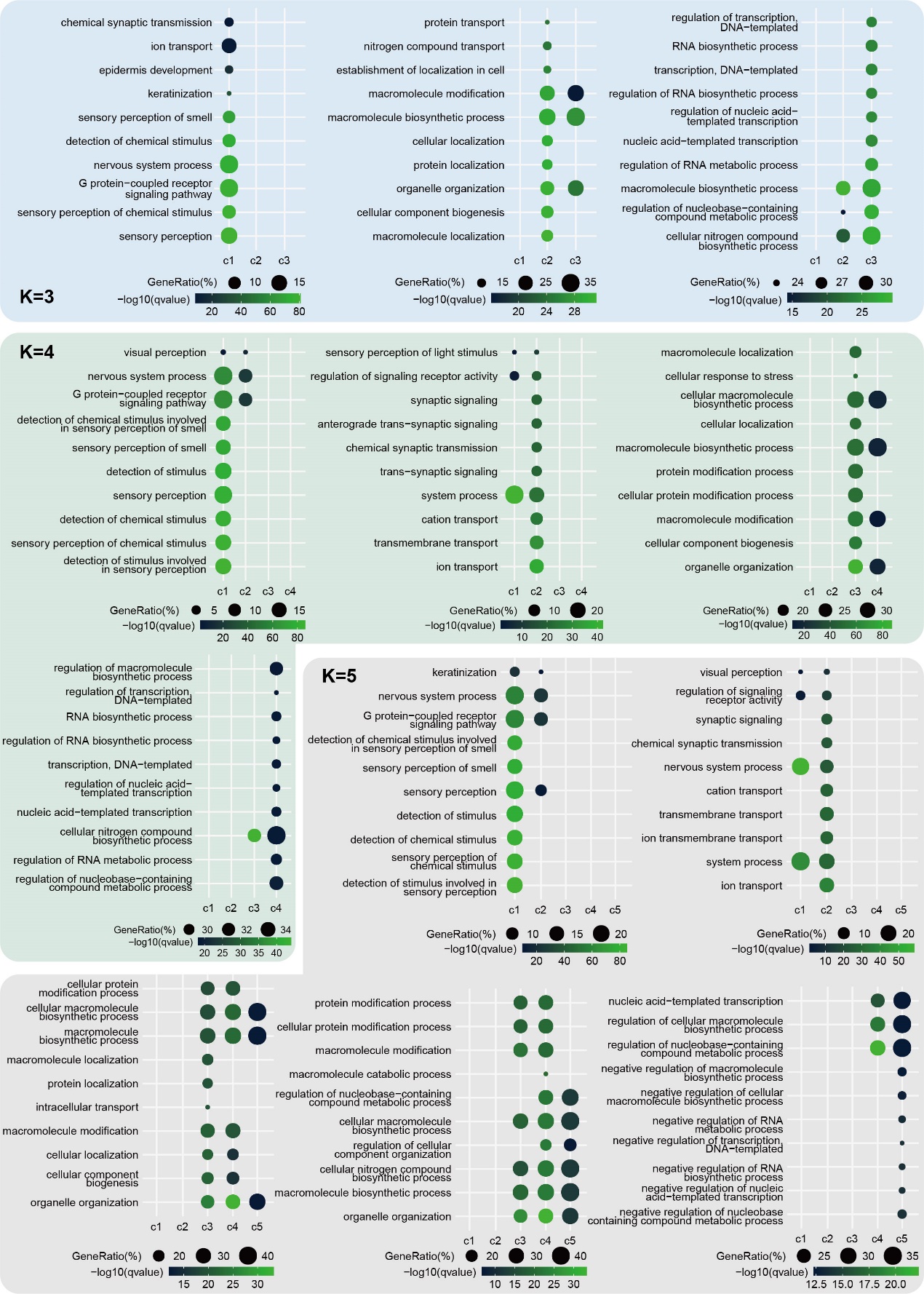
 Fig. S4 GO enrichment analysis of gene clusters classified at different k values.**

**Fig. S5**

**
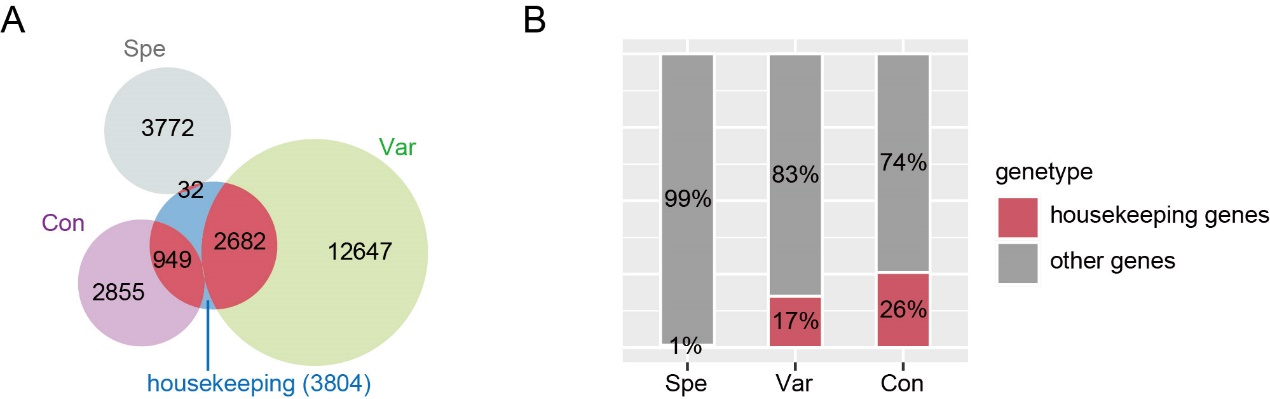
**

**Fig. S5. Proportion of housekeeping genes for Spe, Var and Con patterns.** (**A**) Overlap of housekeeping genes and genes of three regulation patterns. (**B**) Proportion of genes classified as housekeeping genes for three regulation patterns.

**Fig. S6**

**
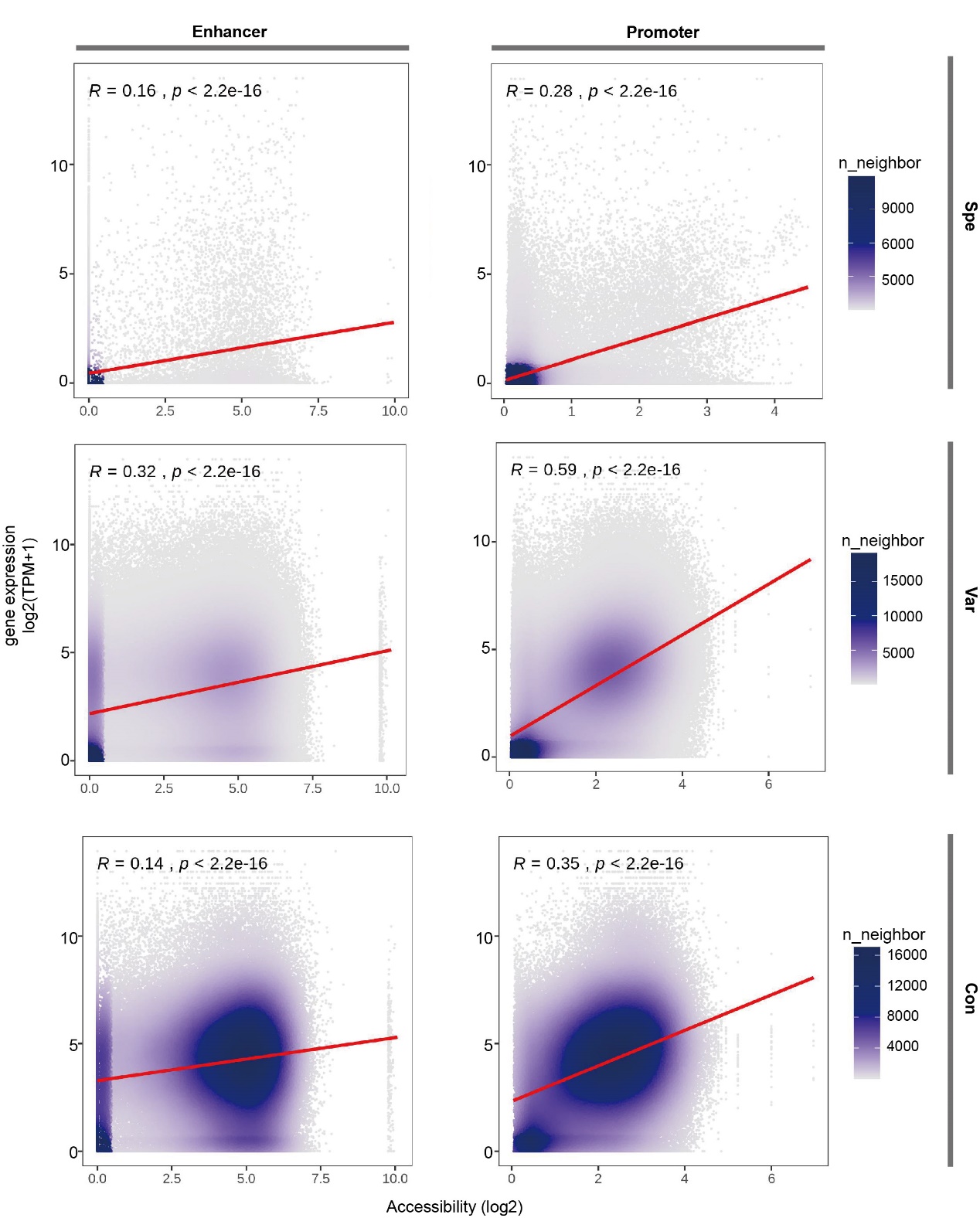
**

**Fig. S6 Spearman correlation between gene expression and accessibility of enhancers and promoter for Spe, Var and Con patterns.** The accessibility was measured by ATAC-seq or DNase-seq (if ATAC-seq data is unavailable)**.** The *x*-axis represents the expression of each gene in each cell, while the *y*-axis represents the accessibility of the promoter corresponding to each gene in each cell and the accessibility of the connected enhancers. The color bar indicated the density of the points. The enhancer accessibility was the sum of accessibility of all enhancers linked to one gene in each cell.

**Fig. S7**

**
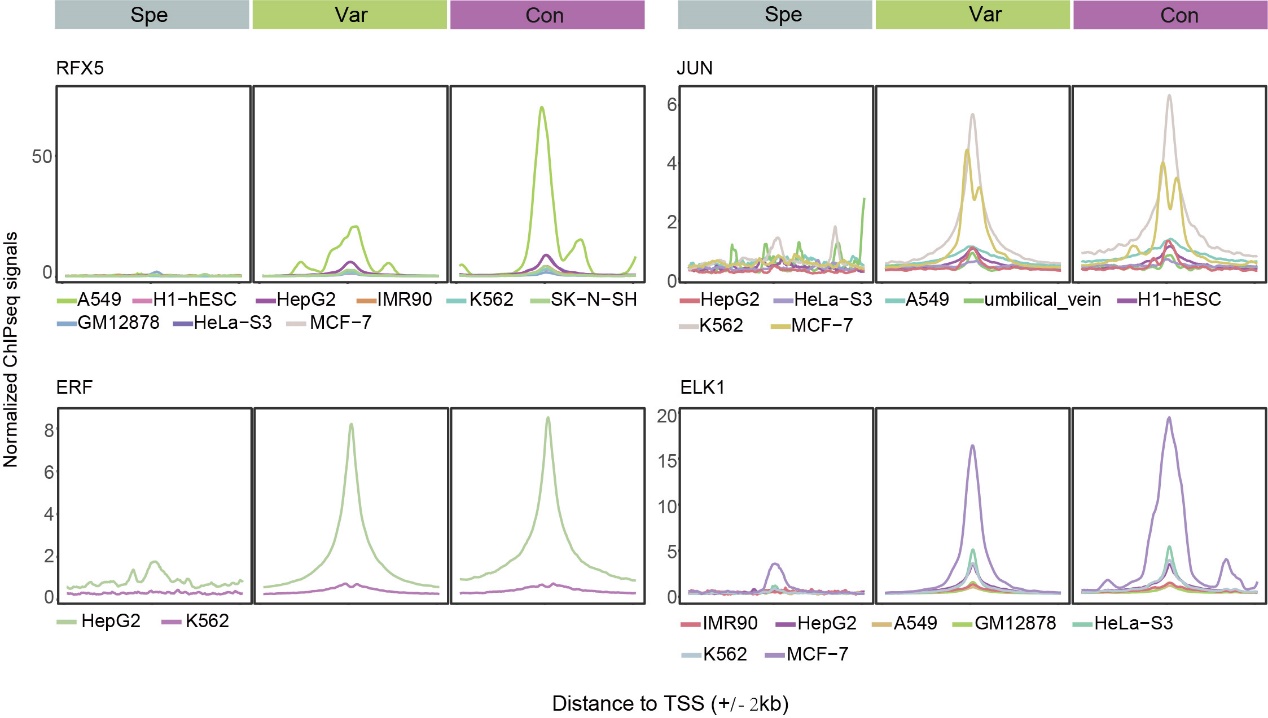
**

**Fig. S7 The signal intensity of more selected TF at promoters for Spe, Var and Con patterns.** The *x*-axis represents the region of TSS ±2kb.

**Fig. S8**

**
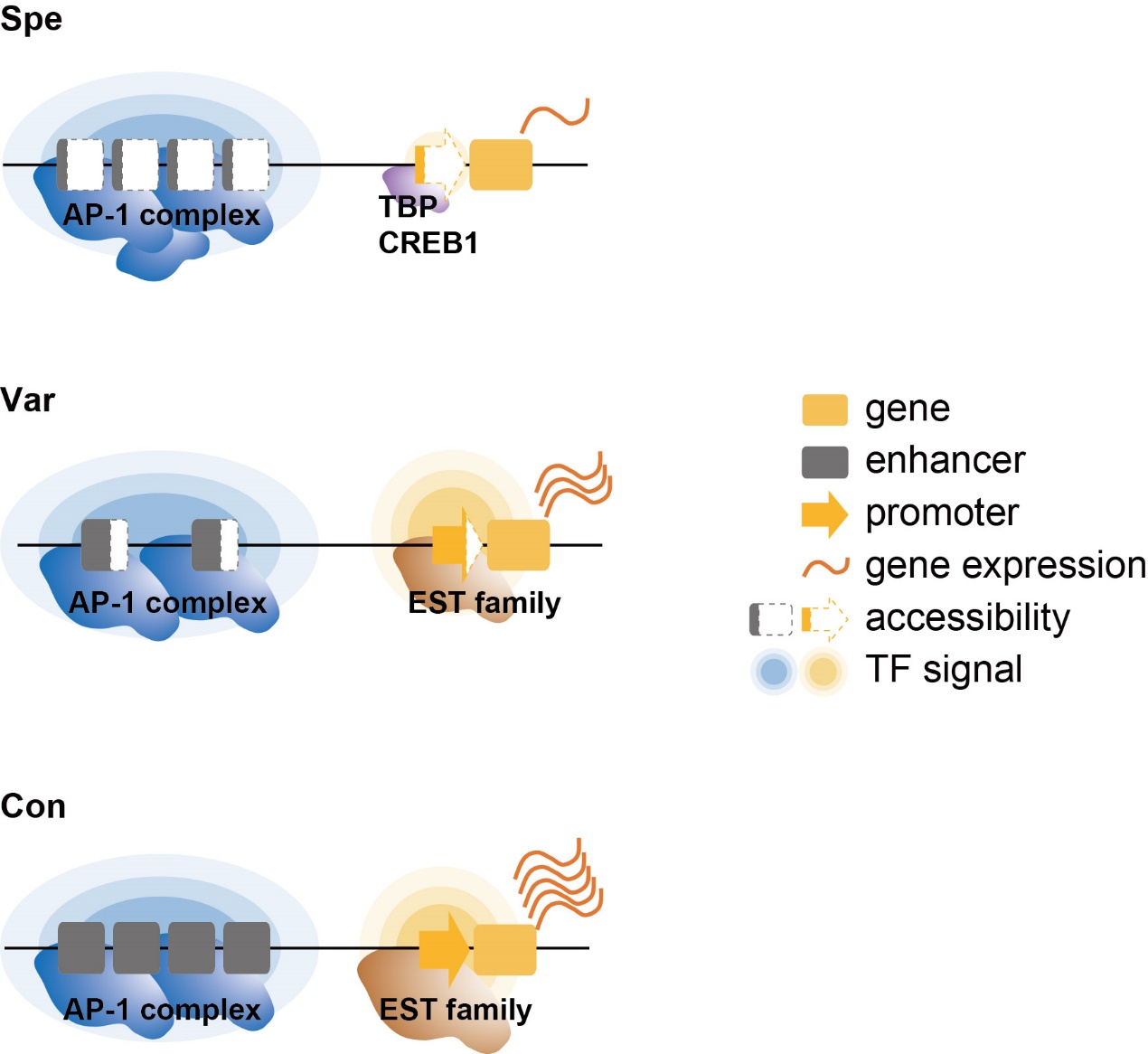
**

**Fig. S8 A model delineating the epigenetic features and potential regulatory roles of promoters and enhancers for Spe, Var and Con patterns.** The accessibility of promoters and enhancers of Spe was the lowest, followed by Var, and Con was the highest. Three different types of enhancers showed similar motif enrichment and TF signal intensity, mainly AP-1. Weakly accessible promoters (Spe) preferred binding with TBP and CREB1, while strongly accessible promoters (Var and Con) prefer binding with the EST family.

**Fig. S9**

**
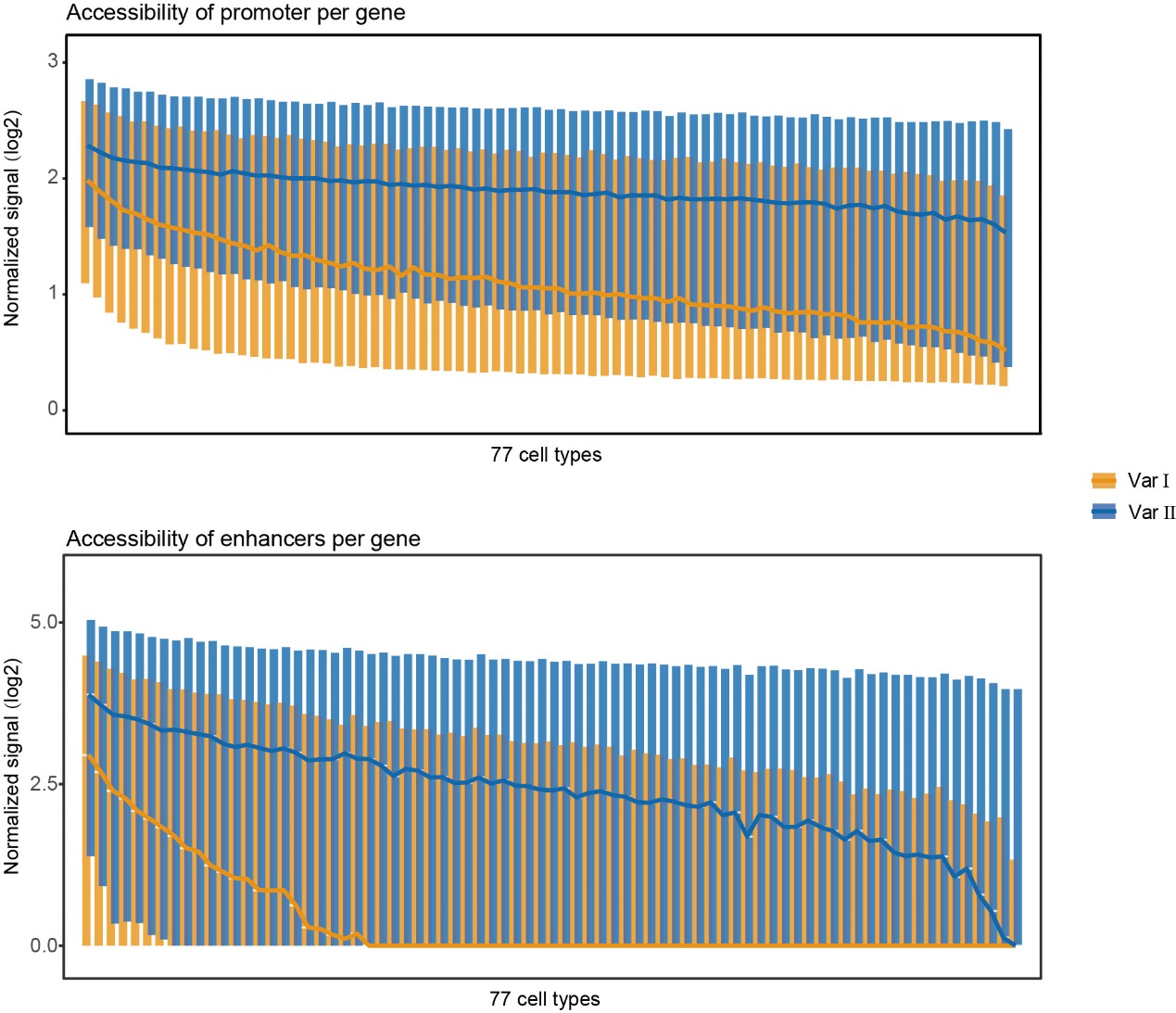
**

**Fig. S9 Chromatin accessibility of promoters and enhancers for each Var gene across 77 cell types.** Enhancer accessibility indicated the sum of all enhancer accessibility for each Var gene. The *x*-axis order is consistent with the **Fig. 5D**. Chromatin accessibility was measured by ATAC-seq or DNase-seq (if ATAC-seq data is unavailable).

**Fig. S10**

**
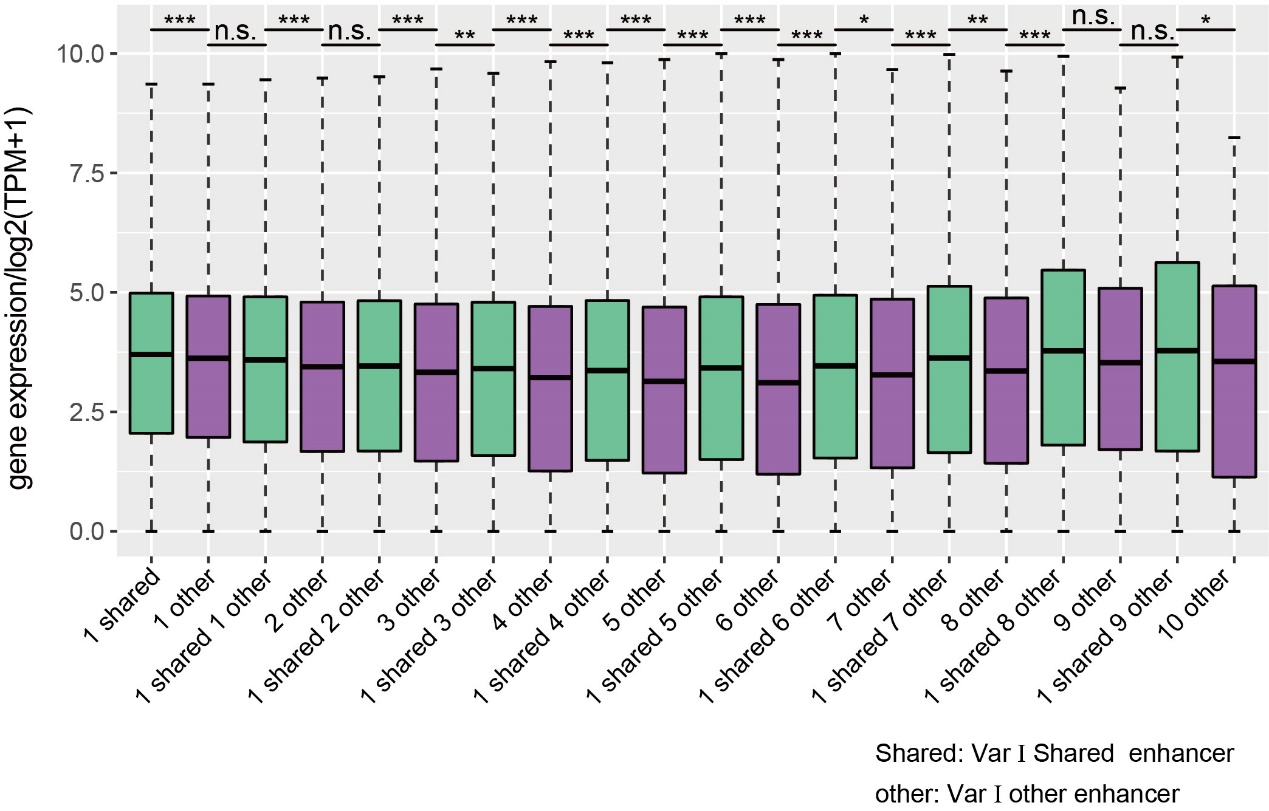
**

**Fig. S10 The expression of Var I genes in cell types with different numbers of Shared enhancer regulation.** Shared enhancers regulated gene in most cell types, so the green boxes indicated gene expression in cell types without Shared enhancer regulation. For example, if the Shared enhancer of gene g was present in 60 cell types, the gene expression of the remaining 17 cell types was counted in the group with only other cell specific enhancers. P-values were calculated using Wilcoxon test. **p* < 0.05; ***p* < 0.01; ****p* < 0.001; *n.s.*, not significant.

**Fig. S11**

**
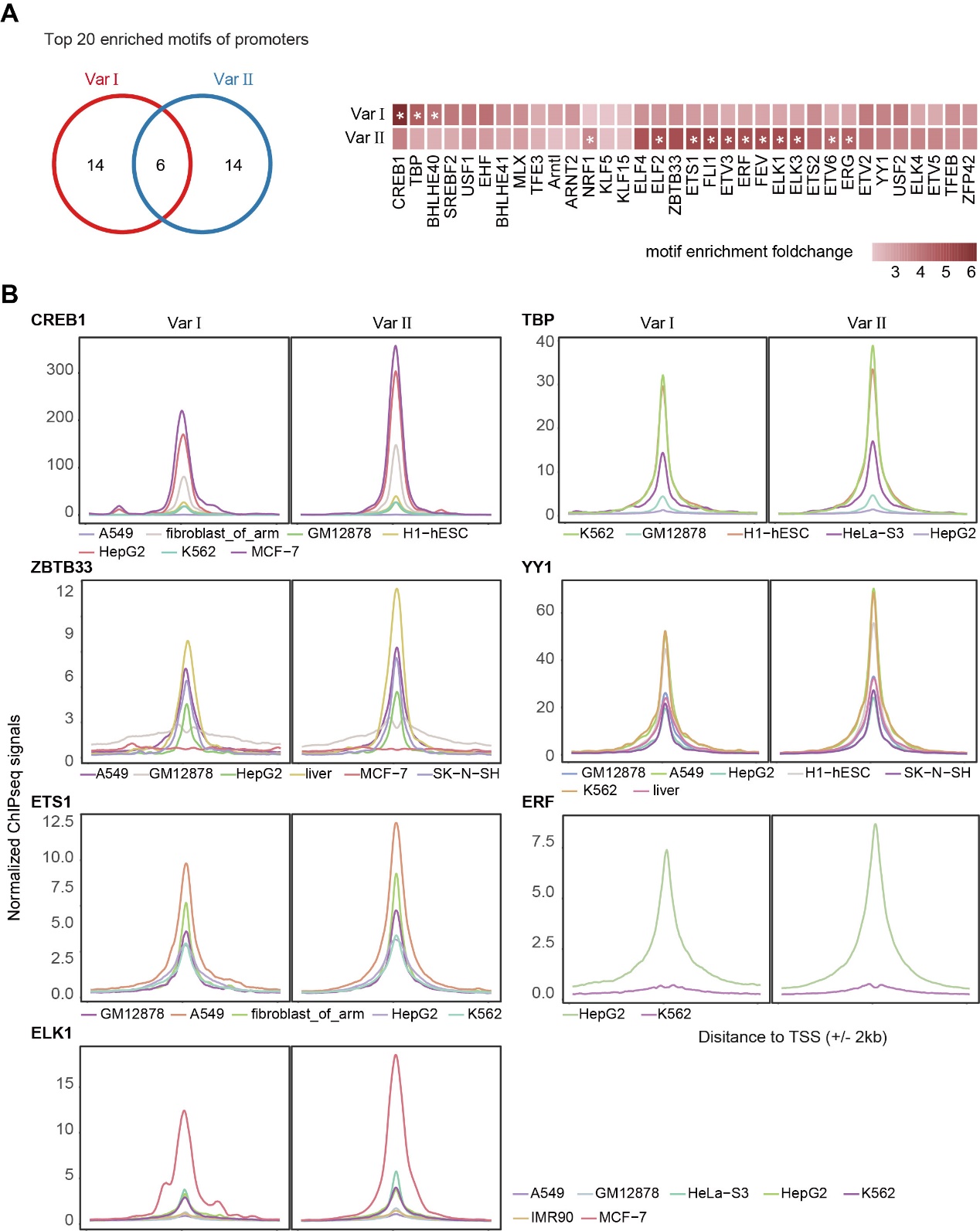
**

**Fig. S11 Motif enrichment analysis and TF ChIP-seq signal for promoters of Var I and Var II.** (A) Motif enrichment analysis. The Veen plot and heatmap showed the overlap and fold-enrichment of the top 20 enriched motifs ranked by q-value in Var promoters, respectively. The asterisk indicated that the motifs showed more than 1.5-fold enriched in this class of promoters than the other one. (**B**) The TF ChIP-seq signal intensity for different Var promoters in various cell types. The *x*-axis represents the region of TSS ±2kb.

**Fig. S12**


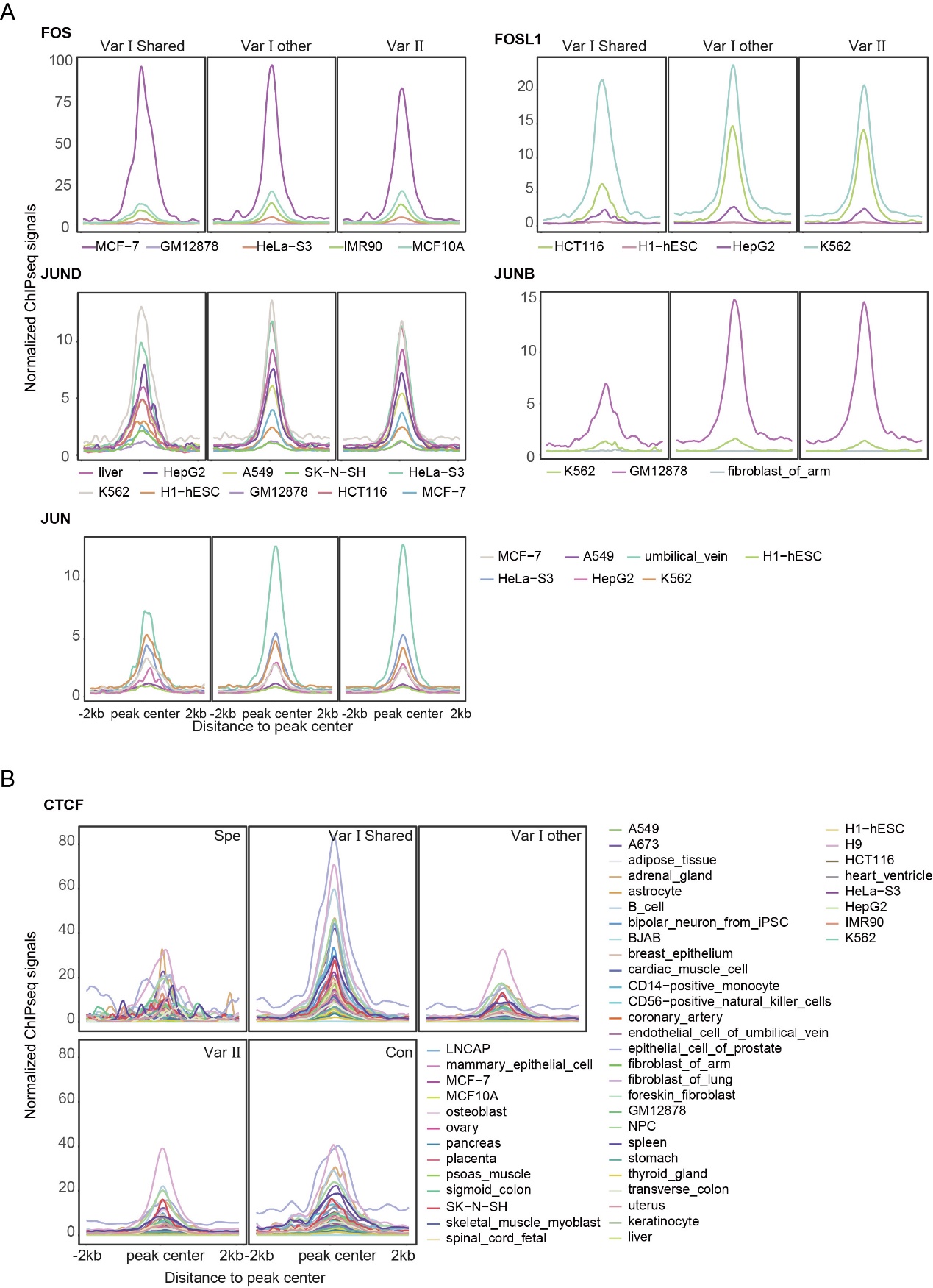


**Fig. S12 The ChIP-seq signal intensity of AP-1 and CTCF for different enhancers in various cell types.** (A) The ChIP-seq signal intensity of more AP-1 family members for different Var enhancers in various cell types. (B) The CTCF signal intensity for enhancers in all patterns. The x-axis represents the region within ±2kb of TSS.
